# A comorbidity of ulcerative colitis, ankylosing spondylitis, Takayasu’s arteritis and silent thyroiditis: an extensive case-based review

**DOI:** 10.1097/MS9.0000000000002495

**Published:** 2024-08-22

**Authors:** Mike Ghabally, George Roumieh, Khaled Qadabashi, Jessica Dayekh, Esber Baydoun, Yusef Jondiah, Besher Shami, Ziena jriekh

**Affiliations:** a Department of Internal Medicine, Aleppo University Hospital; b Department of Radiology, Faculty of Medicine, University of Aleppo, Aleppo, Syria; c AmsterdamUMC, Amsterdam, Netherlands; d Department of Internal Medicine, American University of Beirut Medical Center, Beirut, Lebanon

**Keywords:** ankylosing spondylitis, infliximab, silent thyroiditis, Takayasu arteritis, ulcerative colitis

## Abstract

**Introduction and importance:**

Ulcerative colitis is a chronic condition characterized by continuous inflammation of the rectum and colon. Its clinical complications extend beyond the gastrointestinal tract to involve multiple systems, including musculoskeletal, hepatobiliary, cardiovascular, and ocular manifestations. Takayasu arteritis and ankylosing spondylitis are two autoimmune inflammatory disorders that have previously been reported as coexisting conditions associated with ulcerative colitis. is an autoimmune-mediated inflammation of the thyroid gland causing the release of thyroid hormones and is considered a variant form of chronic autoimmune thyroiditis (Hashimoto’s thyroiditis).

**Case presentation:**

The authors report a case of a 32-year-old Caucasian man with a 3-year history of ulcerative colitis who presented to our clinic in 2015, complaining of chronic lower back pain that alleviates with exercise but does not relieve with rest. Based on our physical exam findings and lab results, ankylosing spondylitis was diagnosed using Assessment of Spondylarthritis International Society and New York criteria. Computerized tomography angiography was performed and demonstrated stenosis in multiple arteries. These findings satisfied the American College of Rheumatology criteria for Takayasu’s disease. After the Infliximab was started, the patient had significant symptomatic improvement.

**Clinical discussion:**

The use of biological therapy plays a key role in the treatment of many autoimmune illnesses. The patient was considered resistant and nonresponsive to treatment; thus, biological therapy was indicated, and infliximab was administered, resulting in a significant clinical improvement and remission of all illnesses.

**Conclusion:**

The authors aim to report a rare coexistence of ulcerative colitis, ankylosing spondylitis, Takayasu’s arteritis and silent thyroiditis. And the authors believe this can aid in diagnosing and management of rare diseases.

## Background

Highlights

- We are presenting the first case of the comorbidity of Takayasu’s disease, ankylosing spondylitis, ulcerative colitis, and silent thyroiditis. Our search through the medical literature revealed only one case of a Japanese patient who was diagnosed with Takayasu’s disease, ankylosing spondylitis, and ulcerative colitis. The use of biological therapy plays an important role in the treatment of many autoimmune illnesses. Our patient was considered resistant and nonresponsive to treatment; thus, biological therapy was indicated, and infliximab was administered, resulting in a great clinical improvement and remission of all illnesses.

Ulcerative colitis (UC) is a chronic condition characterized by continuous inflammation of the rectum and colon. Its clinical complications extend beyond the gastrointestinal tract to involve multiple systems, including musculoskeletal, hepatobiliary, cardiovascular, and ocular manifestations^1^.

Takayasu arteritis (TA) and ankylosing spondylitis (AS) are two autoimmune inflammatory disorders that have previously been reported as coexisting conditions associated with ulcerative colitis^2,3^.

Silent thyroiditis, also known as painless thyroiditis, is characterized by an autoimmune-mediated lymphocytic inflammation of the thyroid gland resulting in destructive thyroiditis with the release of thyroid hormone and transient thyrotoxicosis (hyperthyroidism). This is frequently followed by a hypothyroid phase before recovery of normal thyroid function^4^.

In this manuscript, we present a case of a 32-year-old Caucasian male with a history of ulcerative colitis who concurrently developed Takayasu arteritis and ankylosing spondylitis and was complicated by a cerebellar stroke and silent thyroiditis. Simultaneous occurrence of Takayasu arteritis, ankylosing spondylitis, and ulcerative colitis is extremely rare and has only been reported in one Japanese patient^5^. We believe that our case presents an infrequent presentation of an already rare condition.

## Case report

**Figure 1:**
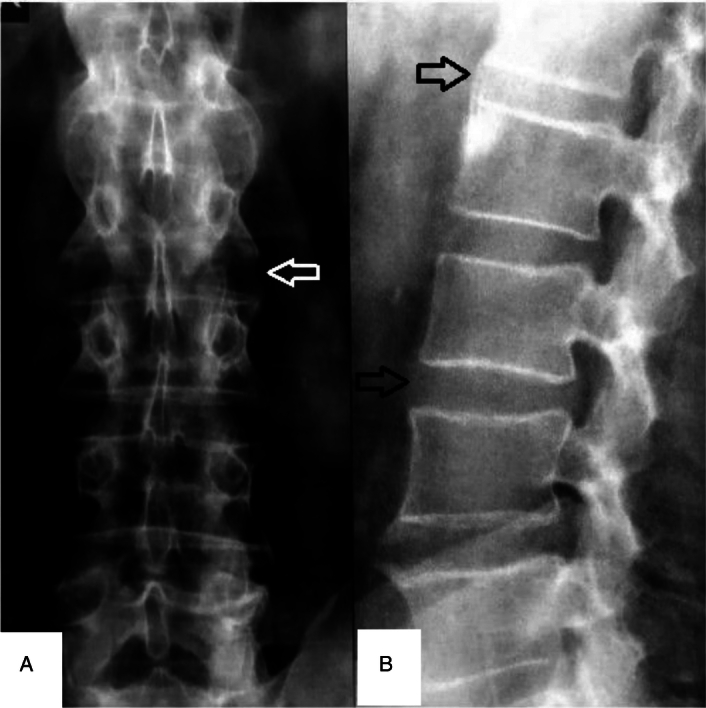
Spinal X-ray demonstrated bilateral grade II sacroiliitis and loss of lumbar lordosis (A, B).

A 32-year-old Caucasian man with a 3-year history of UC presented to our clinic in 2015, complaining of chronic inflammatory lower back pain that improves with exercise but does not relieve with rest. The patient also described morning stiffness and bilateral buttock, bilateral hip, left shoulder, and left ankle pain that can be relieved with the use of non-steroidal anti-inflammatory drugs (NSAIDs). In addition, the patient mentioned night sweats, low-grade fever, and left arm claudication. Physical examination was positive for limited spinal mobility, pulseless left arm arteries with immeasurable blood pressure, weak radial pulse in the right arm with a blood pressure of 100/82 mmHg. However, femoral, popliteal, and dorsalis pedis pulses were easily palpable. Also, Schober’s test was positive. On auscultation, a carotid bruit was audible over the left sternocleidomastoid muscle. Laboratory tests are shown in (Table 1). Spinal X-ray demonstrated bilateral grade II sacroiliitis and loss of lumbar lordosis (Fig. 1). Therefore, AS was diagnosed using the Assessment of Spondylarthritis International Society (ASAS) and New York criteria.

**Table 1:** Laboratory findings

	2012 (UC)	2015 (AS + TA)	2022 (UC flare)	2023 (on infliximab)
Hematocrit	33%	31%	31.9%	38%
RBC (c/ ${\mathrm{mm}}^{3})$	4.6	4.2	4.91	4.15
Hemoglobin (g/dl)	9.4	8.3	8.1	12.8
MCV (Fl)	69	67.3	65	72
WBC (/ ${\mathrm{mm}}^{3})$	12 800	11 100	9200	6870
Platelets count	606 000	486 000	511 000	387 000
ESR (mm/h)	—	30	70	15
CRP (mg/l)	85	131.7	11.4	6.1
C3 (mg/dl)	—	179	—	—
C4 (mg/dl)	—	52	—	—
Prothrombin time (sec)	—	15.2	15.0	13.0
Prothrombin activity	—	65%	71%	94%
INR	—	1.32	1.2	1
Iron (ug/dl)	12	20	35	—
RF/anti CCP	—	Negative	Negative	—
ANA	—	Negative	Negative	—
C-ANCA/P-ANCA	—	Negative	Negative	—
FOBT	Positive	—	Positive	Negative
Protein electrophoresis:				
Albumin	47%–37.25 g/dl	—	42.8%–34.7 g/dl	—
Alpha-1	8.3%–6.56 g/dl	—	10.0%–8.1 g/dl	—
Alpha-2	19.1%–15.09 g/dl	—	19.0%–15.4 g/dl	—
Beta-1	5.2%–4.11 g/dl	—	5.8%–4.7 g/dl	—
Beta-2	6.3%–4.98 g/dl	—	5.4%–4.1 g/dl	—
Gamma	13.9%–0.98 g/dl	—	17.0%–13.4 g/dl	—
TSH	—	—	0.17 uIU/ml	2.8 uIU/ml
T4	—	—	0.766 ng/dl	1.322 ng/dl
T3	—	—	20.43 pmol/l	5.42 pmol/l

ANCA, antineutrophil cytoplasmic antibodies; CCP, cyclic citrullinated peptide; CRP, C-reactive protein; ESR, erythrocyte sedimentation rate; FOBT, fecal occult blood test; INR, international normalised ratio; MCV, mean corpuscular volume; RBC, red blood cell; RF, rheumatoid factor; TSH, thyroid-stimulating hormone; WBC, white blood cell.

**Figure 2:**
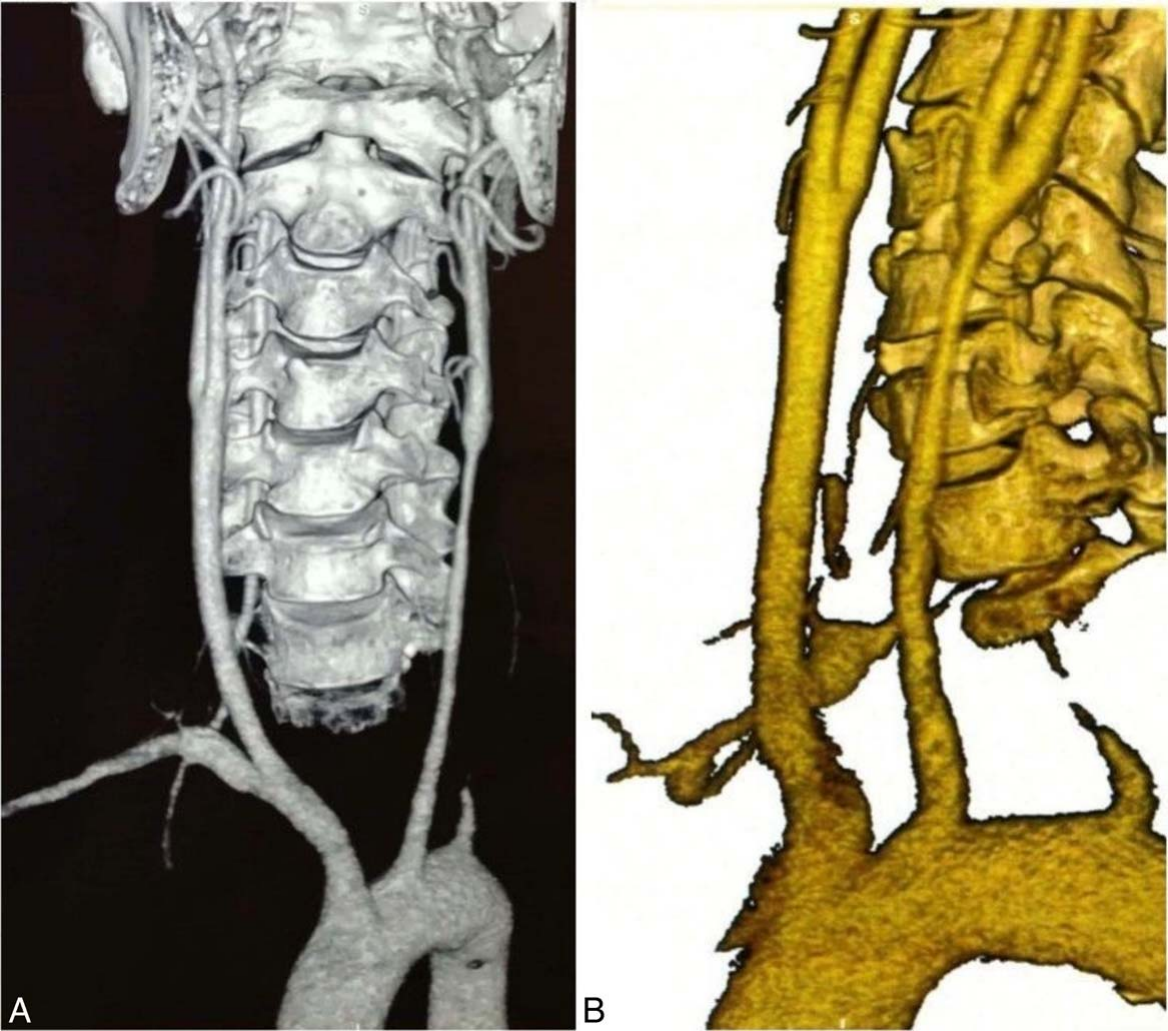
Computed tomography angiography demonstrating stenosis in the left common carotid artery, left vertebral artery, right subclavian artery, and occlusion of the left subclavian artery (A, B).

In addition, TA was suspected, therefore, arterial Doppler ultrasonography was ordered, and it revealed stenosis of the left common carotid and subclavian arteries. Computerized tomography (CT) angiography was performed and demonstrated stenosis in the left common carotid artery, left vertebral artery, right subclavian artery, and occlusion of the left subclavian artery (Fig. 2). These findings satisfied the American College of Rheumatology (ACR) criteria for TA diagnosis^5^. Consequently, Prednisolone 40 mg/day was prescribed and resulted in symptomatic improvement.

**Figure 3:**
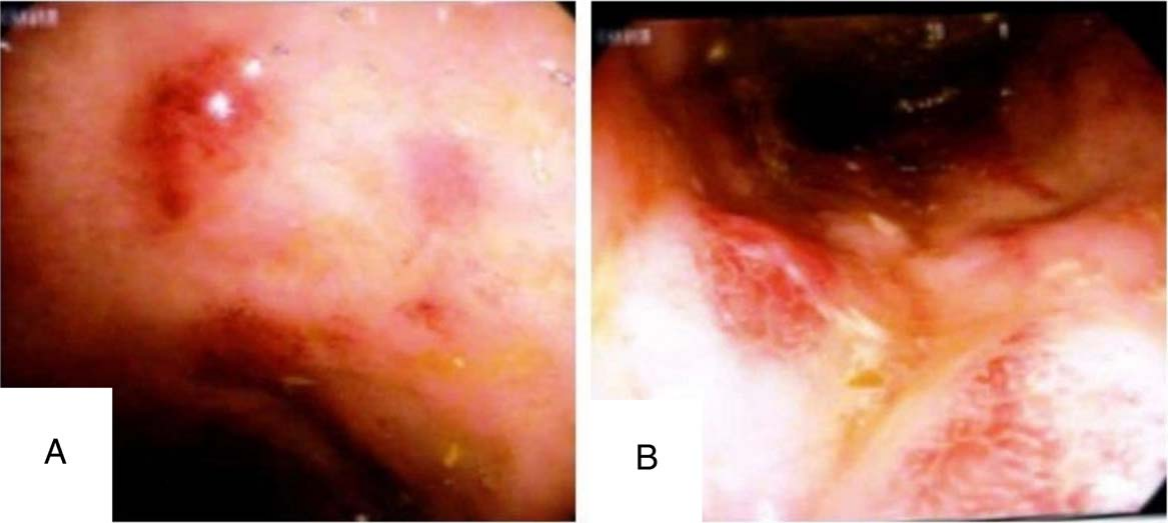
Colonoscopy revealing edematous, erythematous, friable mucosa with surface ulcerations extending from the rectum to the splenic flexure of the colon (A, B).

The patient’s past medical history of UC started in 2012 (at the age of 29) when he experienced severe diarrhea, weight loss of 12 kg in 6 months (94–82 kg), and abdominal pain. Laboratory tests revealed iron-deficiency anemia (Table 1), and stool specimens were positive for occult blood. Colonoscopy revealed edematous, erythematous, friable mucosa with surface ulcerations extending from the rectum to the splenic flexure of the colon (Fig. 3). Furthermore, colonic biopsies confirmed the diagnosis of UC, and prednisolone 20 mg/day and mesalazine 4 g/day were prescribed. However, the patient was non-compliant with medications. The patient has no significant family history.

**Figure 4:**
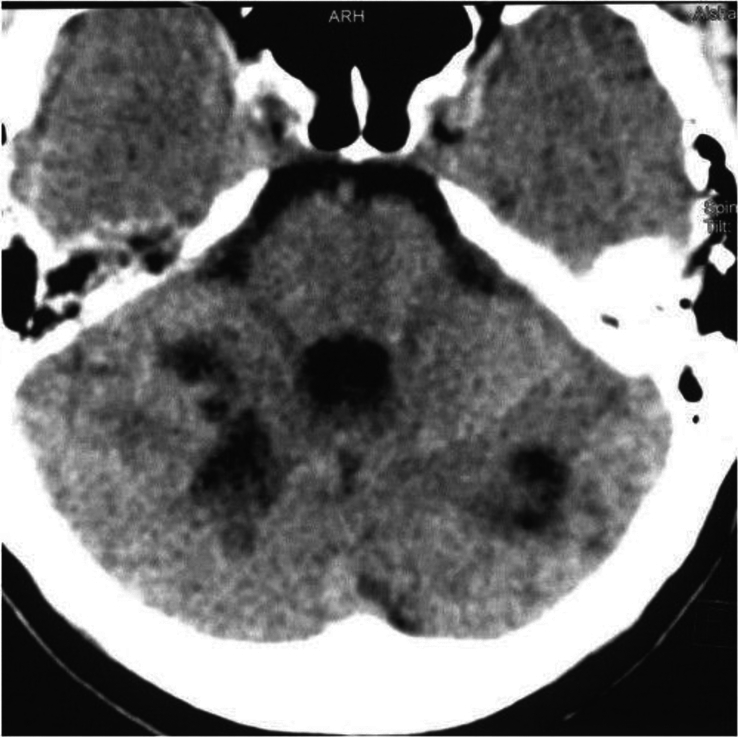
Brain computed tomography scan showing low-density areas located in both cerebellar hemispheres, indicative of cerebellar infarction.

In 2019, at the age of 36, the patient presented to the emergency department with a sudden loss of consciousness, dysarthria, slurred speech, and blurred vision. A brain CT scan showed low-density areas located in both cerebellar hemispheres, indicative of cerebellar infarctions (Fig. 4). Aspirin was administered, and prednisolone was tapered to 5 mg/day. A few months later, the patient made a good recovery with minimal cerebellar complications.

**Figure 5:**
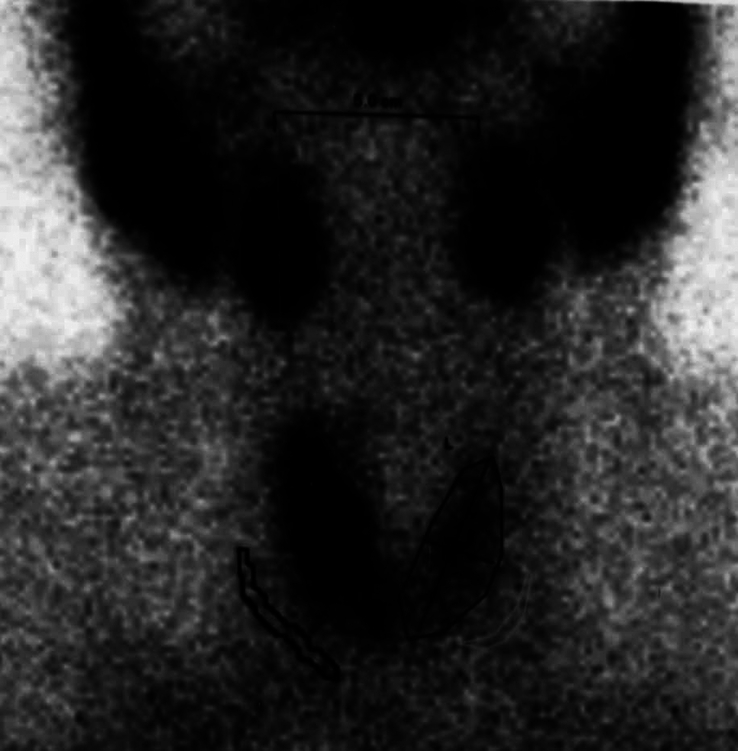
Ultrasonography and 99mTC pertechnetate thyroid scintigraphy.

**Figure 6:**
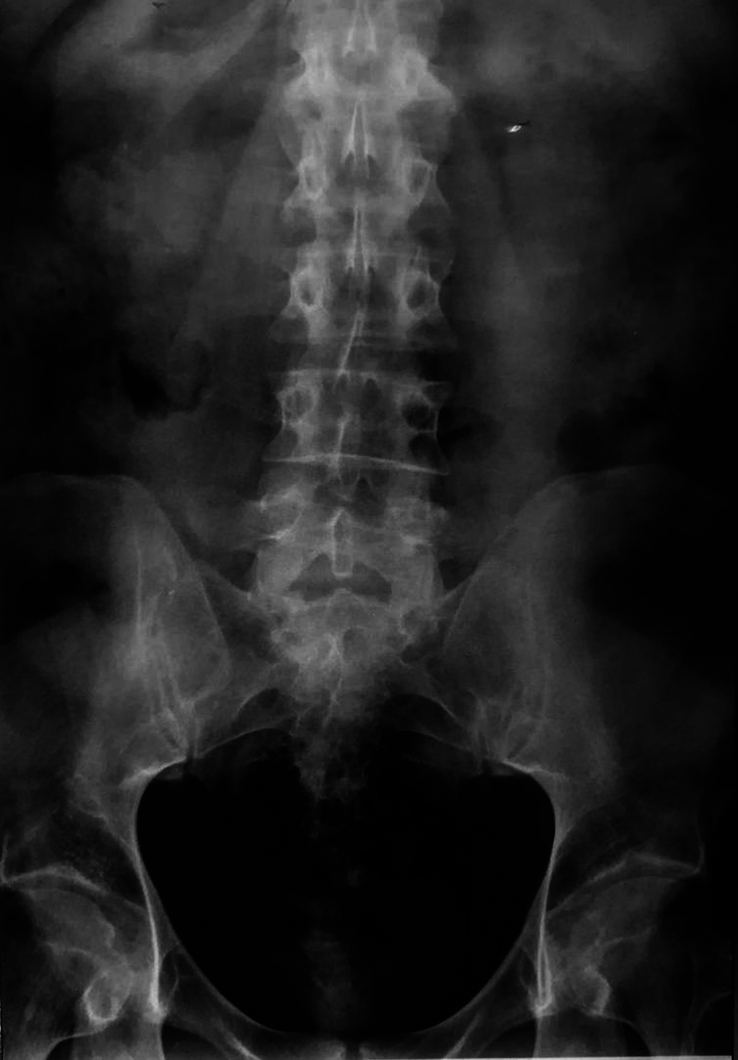
Pelvic X-ray showed bilateral grade IV sacroiliitis.

In 2022, at the age of 39, the patient developed painless transfer dysphagia due to thyroid enlargement. Although there was no clinical evidence of thyroid dysfunction, laboratory findings were suggestive of silent thyroiditis (Table 1). Ultrasonography and 99mTC pertechnetate thyroid scintigraphy were performed, and the patient was diagnosed with silent thyroiditis (Fig. 5). Subsequently, his complaint was resolved spontaneously within 3 months. Later, the patient experienced a new flare of UC, and pelvic radiography showed bilateral grade IV sacroiliitis (Fig. 6). At this point, his condition looked relapsing and refractory, which necessitated treatment modification. Azathioprine was discontinued, and infliximab was started. After the 9th injection of infliximab, the patient had significant symptomatic improvement with regression of arm claudication and back pain and recovered normal bowel habits with a weight gain of 8 kg. Furthermore, C-reactive protein and erythrocyte sedimentation rate levels decreased to 6.1 mg/L and 15 mm/h, respectively.

## Discussion

Ulcerative colitis (UC), ankylosing spondylitis (AS), and Takayasu’s arteritis (TA) are three diseases of idiopathic etiology. AS is more common in white people with an incidence of 1–2% and a female-to-male ratio of 2:1^3^. Similarly, UC is more commonly seen in the white population of North America and Europe^6^. However, TA is more frequent in individuals from Japan, Southeast Asia, and Africa, especially women under the age of 40 years^6,7^.

Given this ethnic distribution, one would expect these three disorders to rarely occur concomitantly. Nevertheless, we believe that our patient represents the second case to develop an overlap of the three diseases, with additional complications of silent thyroiditis and stroke.

The specific etiology of these associations is debatable in the literature. In individuals with an already compromised immune system, there is accumulating evidence that genetic factors, cytokine profile abnormalities, infectious disorders, and allergic reactions play a substantial predisposing role^1,5,8^.

The state of the vertebrobasilar system and posterior communicating artery are the most important predictors of cerebrovascular events^9^. Our patient’s cerebellar stroke may have been caused by the presence of vertebral stenosis in addition to carotid stenosis, which usually prevented the compensatory dilatation of the vertebral arteries that protects most patients with TA from experiencing cerebrovascular accidents^10^. Thromboembolic phenomena are also known complications of UC that occur in 1.2–6.4% of patients, which may have contributed to this ischemic accident^10^.

Our patient’s anemia might have been due to fecal blood loss, the presence of chronic diseases, and steroid-induced bone marrow suppression. The standard initial treatment for active TA is high-dose prednisolone. In case of treatment failure, conventional immunosuppressive agents can be prescribed, all with equal efficacy^11^. For AS, NSAIDs are the first-line therapy and the mainstay of treatment^2^. Unfortunately, none of the disease-modifying antirheumatic drugs have proven any beneficial role in the treatment of axial AS^1^. However, tumor necrotizing factor alpha (TNF-α) blocking agents have played a significant role in inducing and sustaining remission^1^. For UC, treatment choices include steroids, oral aminosalicylates, mesalazine, and sulfasalazine^12^. The use of biological therapy has been increasingly popular in recent decades, as it plays a key role in the treatment of many autoimmune illnesses. In addition, infliximab is regarded as the drug of choice for people with AS and inflammatory bowel disease^2^. Many TA patients who are resistant to steroids have been observed to benefit from infliximab treatment^13^. Other papers, on the other hand, documented a paradoxical development of autoimmune disorders, including TA, when using infliximab^14^. Our patient was treated with corticosteroids, disease-modifying antirheumatic drugs (DMARDS), and NSAIDs without response. His disease was considered resistant and nonresponsive; thus, infliximab was indicated. With infliximab, our patient saw significant clinical improvement and remission of all three illnesses.

## Conclusion

The overlap of AS, TA, and UC could not be incidental, and further studies are needed to elucidate the accurate pathophysiology. Although infliximab seems to be a promising treatment for patients with either of these disorders, it should be used with caution and be reserved for patients with severe refractory conditions. However, additional research is needed to evaluate its therapeutic role.

## Ethical approval

Our institution does not require ethical approval for reporting individual cases or case series.

## Consent

Written informed consent was obtained from the patient for publication of this case report and accompanying images. A copy of the written consent is available for review by the Editor-in-Chief of this journal on request.

## Source of funding

The authors received no financial support for the research, authorship and publication of this article.

## Author contribution

M.G.: first author, data collection. G.R.: writing the paper, data collection. J.D.: data collection. E.B.: data collection. K.Q.: corresponding author, writing the paper. Y.J.: writing the paper. B.S.: writing the paper. Z.J.: supervision.

## Conflicts of interest disclosure

The authors declare no competing interests

## Research registration unique identifying number (UIN)

Not applicable.

## Guarantor

Mike Ghabally.

## Data availability statement

Not applicable.

## Provenance and peer review

Not commissioned, externally peer-reviewed.
